# How Does Less Unethical Behavior Happen? The Moderating Role of Pay Satisfaction on the Disappearance of the Moral Slippery Slope Effect

**DOI:** 10.1002/pchj.70071

**Published:** 2025-12-17

**Authors:** Ying Wu, Binghai Sun, Liting Fan, Sisi Tan, Honglei Ou, Yishan Lin

**Affiliations:** ^1^ School of Psychology Zhejiang Normal University Jinhua China; ^2^ Intelligent Laboratory of Child and Adolescent Mental Health and Crisis Intervention of Zhejiang Province Jinhua China; ^3^ College of Education Jiaxing University Jiaxing China

**Keywords:** moral balance theory, moral slippery slope effect, pay satisfaction, pay type, unethical behavior

## Abstract

The moral slippery slope effect refers to the phenomenon where, within groups or organizations, the incidence of individual unethical behaviors increases and escalates over time. To systematically identify factors that drive the disappearance of this effect, three studies were conducted using a 20‐round spontaneous deception task. Study 1 compared the trend of the moral slippery slope effect under accumulative versus non‐accumulative pay conditions. Results indicated that the moral slippery slope effect disappeared under accumulative pay but persisted under non‐accumulative pay. Studies 2 and 3 further examined the moderating role of pay satisfaction in the moral slippery slope effect, specifically under accumulative pay. Results revealed that pay satisfaction significantly moderated the relationship between experimental rounds and the moral slippery slope effect: the effect persisted when participants reported low pay satisfaction but disappeared when pay satisfaction was high. Collectively, these findings confirm two key conclusions: (1) accumulative pay is a necessary prerequisite for the disappearance of the moral slippery slope effect; (2) pay satisfaction moderates the disappearance of this effect under accumulative pay. This study provides empirical support for moral balance theory and offers practical implications for organizations: designing accumulative pay systems and aligning pay with employee expectations can effectively prevent moral decline by enhancing pay satisfaction.

## 1 Introduction

Ethical research defines the moral slippery slope effect as a phenomenon where, within groups or organizations, once an individual engages in unethical behavior, they tend to repeat and escalate such behavior rather than discontinue it. Essentially, it captures the process by which small unethical acts gradually accumulate into larger ones within these contexts (Baron et al. 2015; Engelmann and Fehr 2016; Köbis et al. 2017; Lafollette 2005; Du et al. 2023a, 2023b; Tang et al. 2019; Welsh et al. 2015; Anderson et al. 2024). Anderson et al. (2024) recently examined this effect from the perspective of moral character judgment, providing further empirical support for its existence. Individuals who commit immoral acts are more likely to repeat such behavior in the future, as unethical conduct weakens the perpetrator's moral sensitivity, fosters subsequent violations, and thus triggers the moral slippery slope effect.

Studying the moral slippery slope effect holds significant theoretical and practical value. Theoretically, it addresses a gap in moral psychology, where prior work in this field has focused primarily on static moral character judgments. Current research on the moral slippery slope effect reveals that moral evaluation involves predicting temporal changes in behavior (Effron et al. 2015; Garrett et al. 2016). Practically, it first explains real‐world concerns, such as judicial concerns about repeated unethical behaviors escalating to crimes. Second, it informs decisions regarding trust, behavioral norms, and organizational ethics (Tang et al. 2019).

A classic method for investigating the moral slippery slope effect is to use multi‐round experimental tasks to test whether the frequency of unethical behavior increases over time (Welsh et al. 2015; Köbis et al. 2015; Marechal et al. 2017). Unethical behavior is typically measured by participants' lying or cheating. For example, Welsh et al. (2015) conducted a three‐round matrix‐solving task with increasing monetary rewards across rounds. They found that the lying rate gradually rose across rounds, thereby confirming the effect's existence.

While the three‐round design reveals short‐term trends in unethical behavior (Kish‐Gephart et al. 2010), it fails to capture behavioral development over extended periods, thereby limiting the generalizability of the moral slippery slope effect. To address this limitation, two key studies increased task rounds to examine the effect's long‐term trajectory. Garrett et al. (2016) used a multi‐round deception task with a fixed pay amount per round, and upon task completion, one round was randomly selected for payment. Their results showed that participants told significantly more lies as rounds increased. However, Effron et al. (2015) reported conflicting findings: they employed a 20‐round deception task where pay was set for each round, and participants received the total sum of earnings across all rounds. They found that participants did not exhibit the moral slippery slope effect until the final round. Specifically, the lying rate decreased when participants knew many rounds remained. However, it increased when they realized the rounds were ending and that they had one final opportunity. These findings reflect the dynamic trend of the moral slippery slope effect and directly contradict the conventional understanding of this effect.

The divergent findings of these two studies highlight a critical distinction: pay type. In Garrett et al. (2016), pay was non‐accumulative and participants received payment from one randomly selected round, whereas in Effron et al. (2015), pay was accumulative and participants received the total earnings across all 20 rounds. This discrepancy is not accidental: subsequent empirical studies have confirmed the moderating role of accumulative pay on unethical behavior (Schmidt et al. 2019; Galliera 2018). Xu et al. (2020), in a behavioral decision‐making study, highlighted that pay type is a key variable shaping individuals' unethical behavior. The “random payment” of non‐accumulative pay creates earnings uncertainty, prompting participants to engage in riskier unethical acts to pursue higher short‐term gains.

Nisan's Moral Balance Theory (Nisan and Horenczyk 1990; Nisan 1991) posits that individuals' moral standards are flexible, shaped by their moral self‐perception and moral balance point. Specifically, when facing conflicts between self‐interest and morality, individuals may engage in moderate unethical actions but adjust their behavior to maintain equilibrium (Markiewicz and Gawryluk 2020; Monin and Jordan 2009; Miller and Effron 2010; Li et al. 2022; Zhou et al. 2025; Perkins et al. 2024). Ultimately, individuals strive to avoid extremes, that is neither “moral saints” nor “moral sinners” (Nisan 1991; Du et al. 2023a, 2023b; Feruglio et al. 2023; Scattolin et al. 2022). For instance, Mazar et al. (2008) demonstrated that participants engaged in moderate lying to secure moderate rewards, rather than maximizing their dishonesty for greater benefits. Fischbacher and Föllmi‐Heusi (2013) documented similar behavior, where participants also restricted the extent to which they resorted to deception. Additionally, empirical research shows that individuals alternate between moral and immoral behaviors based on contextual factors, often choosing more ethical actions once they achieve their intended goals (Song et al. 2021; Zhu et al. 2023).

Under accumulative pay conditions, participants weigh the trade‐off between lying and honesty. The certainty of accumulated earnings alleviates conflicts between self‐interest and morality, allowing participants to achieve “moral balance” and thus engage in less unethical behavior. Over the long term, the incidence of unethical behavior serves as a key indicator of the moral slippery slope effect (Effron et al. 2015; Garrett et al. 2016; Anderson et al. 2024). Based on this, we propose Hypothesis 1:

*Pay type shapes the moral slippery slope effect. Specifically, the effect disappears under accumulative pay but persists under non‐accumulative pay*.

Effron et al. (2015) suggested that accumulative pay enhances individuals' satisfaction with their earnings, which serves as a potential mechanism underlying the disappearance of the moral slippery slope effect. This aligns with Simon's (1978) Principle of Satisfaction, which posits that decision‐making does not pursue rational optimal outcomes but stops once expectations are met (i.e., satisficing), shaped by conventions and norms. Empirical studies confirm a negative correlation between pay satisfaction and unethical behavior (Tang and Chiu 2003). Hofmann et al. (2009) found that employees with high pay satisfaction exhibited significantly fewer unethical behaviors. Veldhuizen (2013) reported that only 38% of high‐income civil servants accepted bribes, compared to over 91% of low‐income counterparts. These findings demonstrate that individuals weigh costs and benefits to achieve subjective satisfaction when making ethical decisions (Gneezy 2005; Mazar et al. 2008; Schauer and Zeckhauser 2010).

Furthermore, high pay satisfaction strengthens moral behavior and self‐control (Ai et al. 2023; Chen et al. 2014; Liang et al. 2021), while pay dissatisfaction drives more unethical conduct (John et al. 2014). Based on this, we propose Hypothesis 2:

*Pay satisfaction moderates the relationship between rounds and unethical behavior. Specifically, under high pay satisfaction, unethical behavior does not show a sustained upward trend, and the moral slippery slope effect is mitigated*.

**FIGURE 1:**
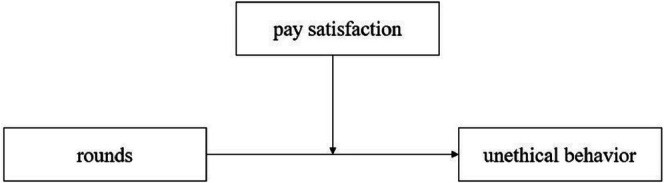
The moderating role of pay satisfaction between rounds and lying rate (a measure of unethical behavior).

Building on the conflicting findings outlined above, as well as relevant theoretical and empirical analyses, this study aims to systematically clarify how pay type (accumulative vs. non‐accumulative) and pay satisfaction shape the moral slippery slope effect. To address these questions, three experiments were conducted, each of which used a 20‐round spontaneous deception task, with two core research questions: First, does pay type (accumulative vs. non‐accumulative) influence the moral slippery slope effect? Second, does pay satisfaction moderate the disappearance of this effect under accumulative pay? Study 1 tests H1 by comparing trends of the moral slippery slope effect under accumulative versus non‐accumulative pay. Studies 2 and 3, conducted exclusively under accumulative pay, test H2 by manipulating pay satisfaction (low vs. high) (see Figure 1). Notably, Study 3 enhances the experiment's ecological validity by aligning pay satisfaction manipulation with participants' ideal pay expectations. We report all data, measures, and experimental conditions in this study. Materials, data, codebooks, and analysis codes are available on the Open Science Framework (OSF) at https://osf.io/zt5f9/?view_only=c2450b42ef764241bea257298322c75b.

## 2 Study 1

### 2.1 Method

#### 2.1.1 Participants

Because the incentive for unethical behavior is often money, this study only recruited college students whose main motivation was to earn money for participation. In the experimental recruitment, we used a preliminary questionnaire survey to understand the participants' motivation to participate. The questionnaire options included: (A) Earning participants' fees, (B) Out of curiosity, and (C) Other. During the screening process, we excluded participants whose record forms were incorrectly filled and whose option was not A. Ultimately, 76 valid participants were included (49 females and 27 males). The average age of the participants was 19.84 years, with a standard deviation of 1.45 years. The participants had normal or corrected‐to‐normal vision, were right‐handed, were not fatigued, and had not previously participated in a similar study. Before conducting the study, the participants filled in the informed consent form.

#### 2.1.2 Study Design

The study adopted a two‐factor mixed design with two pay types (non‐accumulative pay vs. accumulative pay) × 20 rounds, in which pay type was the between‐subjects variable and rounds were the within‐subjects variable. There were 38 participants in each pay type group. The dependent variable was unethical behavior, which manifested as whether participants lied in the dice‐rolling task in each round.

#### 2.1.3 Paradigm

**FIGURE 2:**
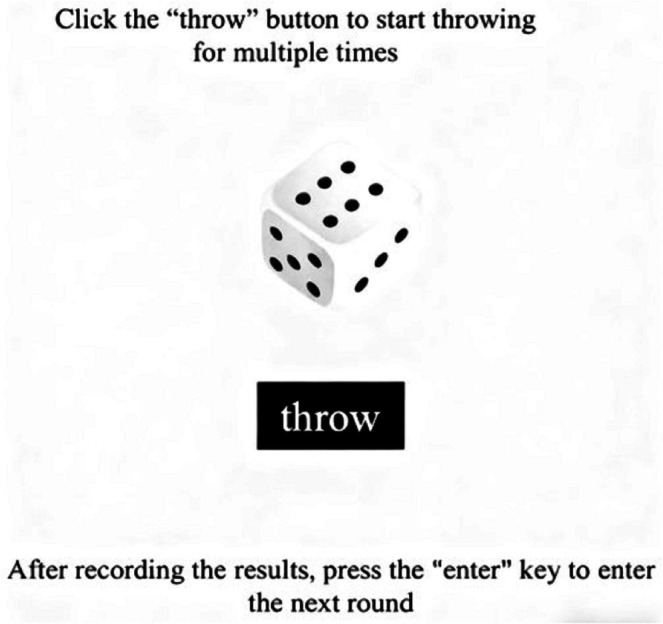
Schematic diagram of study material.

We used the dice‐rolling paradigm for the spontaneous deception task, designed by Fischbacher and Föllmi‐Heusi (2013). Prior to the start of the experimental task, participants were informed that they could click the “throw” button multiple times in each round to throw the dice (Figure 2), but they were required to manually record the result of the first throw on paper (Cao and Niu 2018). We explained to participants that manual recording was necessary due to a supposed programming defect in our study. The program could only store the result of the last throw in each round. This led participants to believe that others would not be aware of the actual outcome of their throws. In this paradigm, the larger the number of dice, the higher the participants' pay. Note that participants' pay was determined based on their manual records. If the number a participant recorded in any round differed from the number stored in the program's backend, the participant was deemed to have lied.

#### 2.1.4 Procedure

Prior to the start of the experiment, participants were provided with experimental instructions. For the non‐accumulative pay condition, the instructions stated: “This study includes 20 rounds. After the experiment, we will randomly select one round, and the reward for that round will serve as your participation fee.” For the accumulative pay condition, the instructions read: “This study includes 20 rounds. After the experiment, your participation fee will be the total sum of pay from all 20 rounds.” Participants' compensation was determined based on their recorded sheets. Once the experimenter confirmed that all participants understood the research process and payment mechanism, they left the room. The entire task was conducted unsupervised, and each participant completed the experiment independently in accordance with the instructions.

After the experiment, participants' pay was calculated as follows: For the non‐accumulative pay condition, pay was based on their recorded sheets, with the seventh round randomly selected as the settlement round. For the accumulative pay condition, the total number of dice points across all rounds was tallied, and the corresponding monetary pay was provided. The experiment was programmed in E‐Prime 3.0. A computer monitor (Dell brand LED display [13.3 in., refresh rate of 60 Hz, resolution of 2560 × 1600]) was used, and participants were seated 50 cm from the screen.

#### 2.1.5 Data Analysis

Sample size and result robustness: As Study 1 was an exploratory study without specific effect size estimates for our research hypothesis, we did not conduct an a priori power analysis. However, results remained stable across experimental conditions (non‐accumulative pay vs. accumulative pay) even with varying participant numbers (≥ 10 per condition), indicating the robustness of the findings (see Supporting Information S1 for consistency across *N* = 10, 16, or 22). A sensitivity power analysis (calculations made using G*Power; Faul et al. 2007) showed that with 76 participants, a minimal effect size of *d* = 0.65 could be detected under standard criteria (*α* = 0.05, 1 − *β* = 0.80, two‐tailed).

Curve fitting was performed on the lying rates for the two pay type conditions using seven nonlinear models, with fitting statistics calculated in SPSS 21.0 (Supporting Information S1).

To further verify the stability of the results, a distributed calculation was performed using MATLAB 13.5. To reflect the group trend of lying behavior, we sampled 38 participants under the conditions of non‐accumulative and accumulative pay 1000 times. Subsequently, the standard deviation of 1000 samples was calculated. To investigate the changing trend in the lying rate with the corresponding rounds, we generated scatter plots for both pay type conditions. Based on the data trends, we fitted the lying rate as a power function of rounds for the non‐accumulative pay condition:

(1) $$\text{Lying rate}=s\times {\text{Round}}^{P},$$

For the accumulative pay condition, the lying rate was fitted as a quadratic function of rounds:

(2) $$\text{Lying rate}=a\times {\text{Round}}^{2}+b\times \text{Rounds},$$

Notably, because the lying rate was zero at round 0 (i.e., no rounds completed), both Equations (1) and (2) were constrained to pass through the origin (coordinates [0,0]). Chi‐square analyses were used to assess the validity of the fitted equations. If the analysis was not significant, the fitting equation was considered valid.

Generalized Estimating Equations (GEEs), developed by Liang and Zeger (1986), are well‐suited for analyzing repeated measurements of participants across time points. GEE models pay type and rounds as separate predictors, such that the interpretation of regression parameters remains unaffected by assumptions about the nature or magnitude of intra‐participant correlations. In our GEE analysis, we included round and pay type as predictors to model intra‐participant effects, with the outcome variable specified as a categorical measure (whether to lie or not).

### 2.2 Results

#### 2.2.1 Model Analysis

**FIGURE 3:**
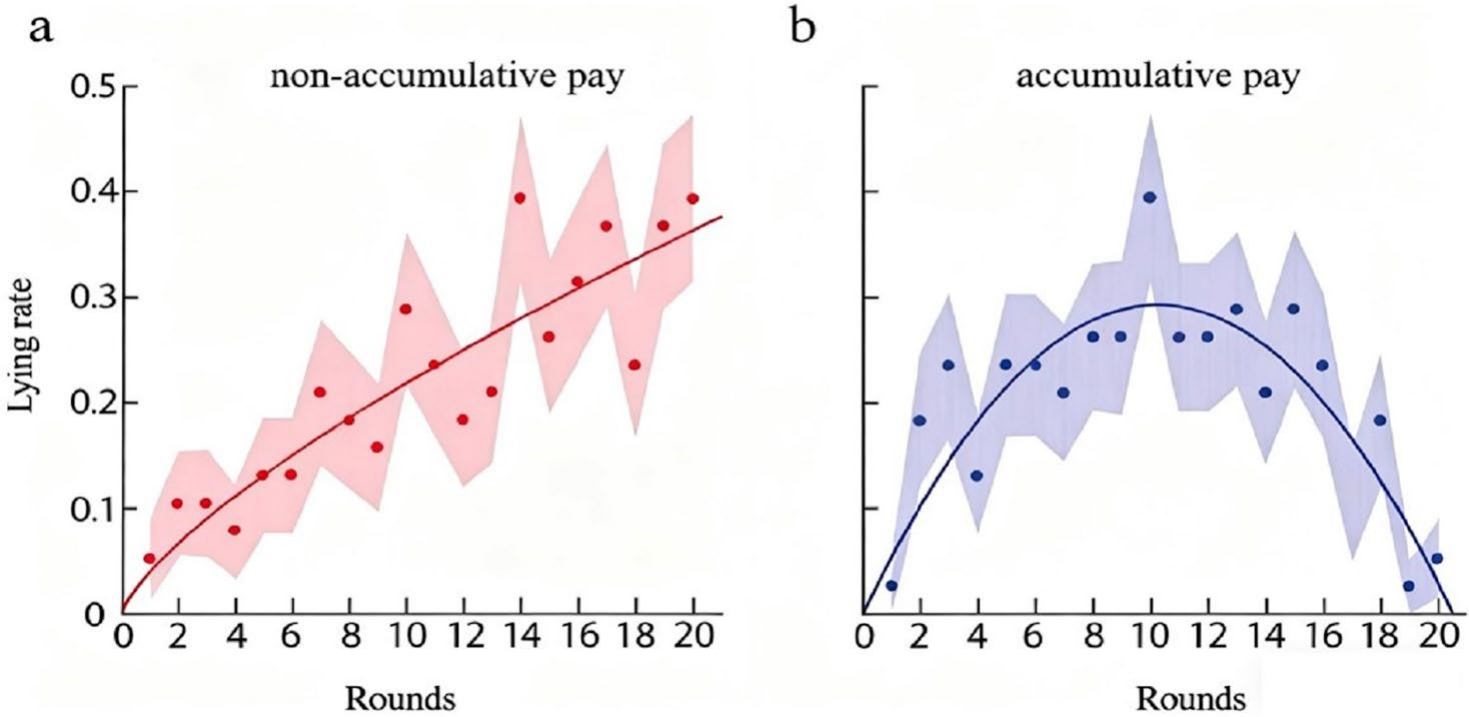
Results of Study 1. Solid dots represent the lying rate across rounds. The shaded area represents the standard deviation of the lying rate after 1000 random samplings. Panel (a) depicts results for the non‐accumulative pay condition, where the lying rate increases with increasing rounds (*R* ^2^ = 0.76, *χ* ^2^_(18)_ = 0.97, *p* = 1.000). Panel (b) depicts results for the accumulative pay condition, where the lying rate first increases and then decreases with increasing rounds (*R* ^2^ = 0.63, *χ* ^2^_(18)_ = 1.02, *p* = 1.000).

Curve‐fitting analyses revealed that for the non‐accumulative pay condition, the power curve and quadratic model were the best‐fitting curves for the “lying rate‐rounds” condition (Supporting Information S1). A further distributed calculation was performed on the power curve under the non‐accumulation pay condition and on the quadratic curve under the accumulation pay condition. Figure 3 illustrates the lying rate trend across rounds. As shown in Figure 3a, the lying rate increased with more rounds in the non‐accumulative pay condition and followed a power function distribution (*R* ^2^ = 0.76, *χ* ^2^_(18)_ = 0.97, *p* = 1.00), indicating the presence of the moral slippery slope effect. Figure 3b shows that the lying rate first increased and then decreased with more rounds in the accumulative pay condition, following a quadratic distribution (*R* ^2^ = 0.63, *χ* ^2^_(18)_ = 1.02, *p* = 1.00), consistent with the disappearance of the moral slippery slope effect.

#### 2.2.2 GEE Analysis

We conducted a GEE analysis to examine how the number of lies varied across rounds for different pay types. Results showed a significant main effect of round (Wald *χ* ^2^_(19)_ = 61.23, *p* < 0.001), but no significant main effect of pay type (Wald *χ* ^2^_(1)_ = 0.12, *p* = 0.725). Importantly, a significant interaction emerged between pay type and round (Wald *χ* ^2^_(19)_ = 61.09, *p* < 0.001). Post hoc simple effect analyses further indicated that, compared to the accumulative pay condition, the measured lying frequency in the non‐accumulative pay condition differed more from that in the first round as the experiment progressed (Supporting Information S1).

### 2.3 Discussion

Study 1 employed the spontaneous deception paradigm and a 20‐round dice‐rolling task to examine how pay type shapes trajectories of unethical behavior. The results supported our initial hypothesis regarding pay type's impact on the moral slippery slope effect. Under non‐accumulative pay, where earnings depended on one randomly selected round, the lying rate rose steadily with more rounds. This pattern reflected a clear moral slippery slope effect, which was consistent with the findings of Garrett et al. (2016). In contrast, under accumulative pay, where earnings were the total of all rounds, the lying rate first increased and then decreased. Here, the moral slippery slope effect disappeared, aligning with the results reported by Effron et al. (2015).

Notably, Effron et al. (2015) previously proposed that accumulative pay may be associated with higher pay satisfaction. Empirical findings further confirm that pay satisfaction is linked to reduced unethical behavior. Hofmann et al. (2009) found that employees with high pay satisfaction exhibited significantly fewer unethical behaviors. Together, these observations suggest that high pay satisfaction may potentially be a key factor underlying the disappearance of the moral slippery slope effect under accumulative pay.

To investigate this question, Study 2 builds on Study 1 by focusing exclusively on the accumulative pay condition. It examines how pay satisfaction influences the moral slippery slope effect by testing whether satisfaction moderates the relationship between rounds and unethical behavior. This extension is critical for clarifying why accumulative pay eliminates the moral slippery slope effect, as it directly addresses the potential mechanism proposed by Effron et al. (2015).

## 3 Study 2

Building on Study 1, which revealed that the moral slippery slope effect disappears under the accumulative pay condition, Study 2 focused on manipulating different levels of pay satisfaction to examine how pay satisfaction influences the disappearance of this effect.

### 3.1 Method

#### 3.1.1 Participants

Study 2 recruited 130 college students who were primarily motivated by monetary pay for participation. Data collection was stopped once the target sample size was achieved, yielding 110 valid female participants and 20 valid male participants. The mean age of participants was 21.30 years (SD = 1.65). All participants had normal or corrected‐to‐normal vision, were right‐handed, reported no fatigue, and had no prior participation in similar studies. Prior to the study, all participants provided written informed consent.

#### 3.1.2 Study Design

We used a two‐factor mixed design with two pay satisfaction conditions (high vs. low pay satisfaction) × 20 rounds, in which pay satisfaction was the between‐subjects variable and rounds were the within‐subjects variable. There were 65 participants in each pay satisfaction condition. The dependent variable was unethical behavior, operationalized as whether participants lied in the dice‐rolling task during each round.

#### 3.1.3 Paradigm

The experimental paradigm used in Study 2 was identical to that in Study 1.

#### 3.1.4 Procedure

Prior to the experiment, high and low levels of pay satisfaction were manipulated. Participants in the low pay satisfaction condition received no basic pay, whereas those in the high pay satisfaction condition received a basic pay (Chen et al. 2014). The process is as follows. The instruction for the low pay satisfaction condition: “This task uses an accumulative pay structure, so please track the accumulation of your pay throughout the task. Your participation fee will be the total of rewards from the upcoming 20 rounds of the dice‐rolling task” The instruction for the high pay satisfaction condition: “This task uses an accumulative pay structure, so please track the accumulation of your pay throughout the task. You will receive a basic participation fee of 8 Yuan, and you can also earn additional rewards from the upcoming 20 rounds of the dice‐rolling task. Your total participation fee will include both the basic fee and additional rewards.”

The 20‐round task took approximately 20 min to complete. Participants were required to make standardized records in the specified format and calculate their actual pay every five rounds while recording. The effectiveness of pay satisfaction manipulation was tested during the experiment. Every five rounds (0, 5, 10, 15, and 20 rounds), participants were asked to respond to a question within the task program: “What is your current pay satisfaction? (1–7 scale, where ‘1’ indicates the lowest level of satisfaction and ‘7’ indicates the highest level).” The pay satisfaction manipulation was deemed effective if participants in the low pay satisfaction condition reported significantly lower pay satisfaction than those in the high pay satisfaction condition at each time point (Chen et al. 2014).

#### 3.1.5 Data Analysis

Sample size determination: An a priori power analysis (conducted using G*Power; Faul et al. 2007) indicated that a sample size of 68 participants was required to achieve a power of 0.80, assuming a medium effect size of *f* ^2^ = 0.15 (Cohen 1988) for the fit equation involving two experimental conditions (low vs. high pay satisfaction) and an *α* level of 0.05. Data collection ceased when this target was met.

To verify the effectiveness of the pay satisfaction manipulation, we used SPSS 21.0 to perform the Kolmogorov–Smirnov test on self‐reported pay satisfaction data across the two conditions, which revealed that the data did not follow a normal distribution. We therefore conducted a nonparametric Mann–Whitney *U* test. Additionally, we report correlations between lying decisions and self‐reported pay satisfaction.

We tested the normality of the number of lies across the 20 rounds for each pay satisfaction condition and report the ratio of lying frequencies between the high and low pay satisfaction conditions.

The curve‐fitting procedure was identical to that in Study 1 (Supporting Information S2). Further, MATLAB 13.5 is used for the distributed calculation. Specifically, the lying rate across rounds in the low pay satisfaction condition was fitted with a power function (Equation 1), while the lying rate in the high pay satisfaction condition was fitted with a cubic function:

(3) $$\text{Lying rate}=a\times {\text{Rounds}}^{3}+B\times {\text{Rounds}}^{2}+C\times \text{Rounds},$$

In Study 2, the GEE analysis, the intra‐participant effects were based on the round and pay satisfaction conditions, and we specified a categorical outcome variable (number of lies). Additionally, we conducted a GEE model that included rounds (0, 5, 10, 15, and 20) and pay satisfaction conditions as intra‐participant effects, self‐reported pay satisfaction at each stage as the covariate, and the number of lies as the dependent variable.

The moderating role of pay satisfaction on rounds and the lying rate was tested as follows: the lying rate between the various rounds was a continuous variable, as was the interference variable (i.e., pay satisfaction), which was a categorical variable. We first used grouping regression analysis and then used the *Z* test (non‐standardized test; Dixon and Duncan 1975), that is, the non‐standardized coefficients *b*_1_ and *b*_2_ and standard errors se_*b*1_, se_*b*2_ in the following formula:

$$Z=\left(b1-b2\right)/\sqrt{{\mathrm{se}}_{b1}^{2}+{\mathrm{se}}_{b2}^{2}}$$

If *Z* < −1.96, it showed that the pay satisfaction had a significant regulatory effect on the lying rate in the different rounds.

### 3.2 Results

#### 3.2.1 Self‐Reported Pay Satisfaction Manipulation Check

Nonparametric Mann–Whitney *U* tests revealed that self‐reported pay satisfaction differed significantly between the two conditions (*Z*s < −4.704, *p*s < 0.001, *d*s > 1.437). Self‐reported pay satisfaction of the low pay satisfaction condition (0 rounds: *M ±* SD = 2.60 ± 1.59, 5 rounds: *M ±* SD = 3.20 ± 1.38, 10 rounds: *M ±* SD = 3.62 ± 1.13, 15 rounds: *M ±* SD = 3.77 ± 1.31, 20 rounds: *M ±* SD = 4.31 ± 1.20) was consistently lower than that of the high pay satisfaction condition (0 rounds: *M ±* SD = 5.00 ± 1.16, 5 rounds: *M ±* SD = 4.48 ± 1.32, 10 rounds: *M ±* SD = 4.78 ± 1.28, 15 rounds: *M ±* SD = 5.15 ± 1.15, 20 rounds: *M ±* SD = 5.65 ± 1.12). These results confirm that the pay satisfaction manipulation was effective.

A correlation analysis was conducted between the self‐reports of the participants in each condition at rounds 0, 5, 10, 15, and 20 and their lying behaviors at each stage. The results showed that low pay satisfaction reports were significantly correlated with lying behaviors (*r* = 0.19, *p* < 0.001), while high pay satisfaction reports were not significantly correlated with lying behaviors (*r* = 0.41, *p* = 0.465).

#### 3.2.2 Lying Frequency and Normality Tests Across Groups

Kolmogorov–Smirnov tests for normality revealed that the low pay satisfaction group rejected the null hypothesis of normality (*p* = 0.012, *p* < 0.05), whereas the high pay satisfaction group failed to reject the null hypothesis (*p* = 0.090). This indicates that only the low pay satisfaction group deviated from a normal distribution, while the high pay satisfaction group's lying frequency distribution aligned with the expected theoretical distribution. Additionally, a Mann–Whitney *U* test showed a significant difference in total lies across the 20 rounds between the two groups (*U* = 18.000, *p* < 0.001). Specifically, the low pay satisfaction group had a significantly higher lying frequency (*M* = 3.43, SD = 3.84) than the high pay satisfaction group (*M* = 1.51, SD = 2.33).

Lying frequencies of the two groups across the 20 rounds are presented in Table 1. Consistent with the total frequency results, the low pay satisfaction group exhibited a higher overall lying frequency than the high pay satisfaction group. At the single‐round level, the low pay satisfaction group's maximum lying frequency reached 17 times, compared to a maximum of 10 times in the high pay satisfaction group.

**TABLE 1:** Study 2: The lying frequency of the two groups.

Low pay satisfaction group			High pay satisfaction group		
Number of lies	Frequency	Effective percentage	Number of lies	Frequency	Effective percentage
6	2	10.00	2	1	5.00
7	1	5.00	3	4	20.00
8	2	10.00	4	6	30.00
9	1	5.00	5	2	10.00
11	3	15.00	6	4	20.00
12	4	20.00	7	1	5.00
13	3	15.00	9	1	5.00
14	3	15.00	10	1	5.00
17	1	5.00	—	—	—

#### 3.2.3 Model Analysis

**FIGURE 4:**
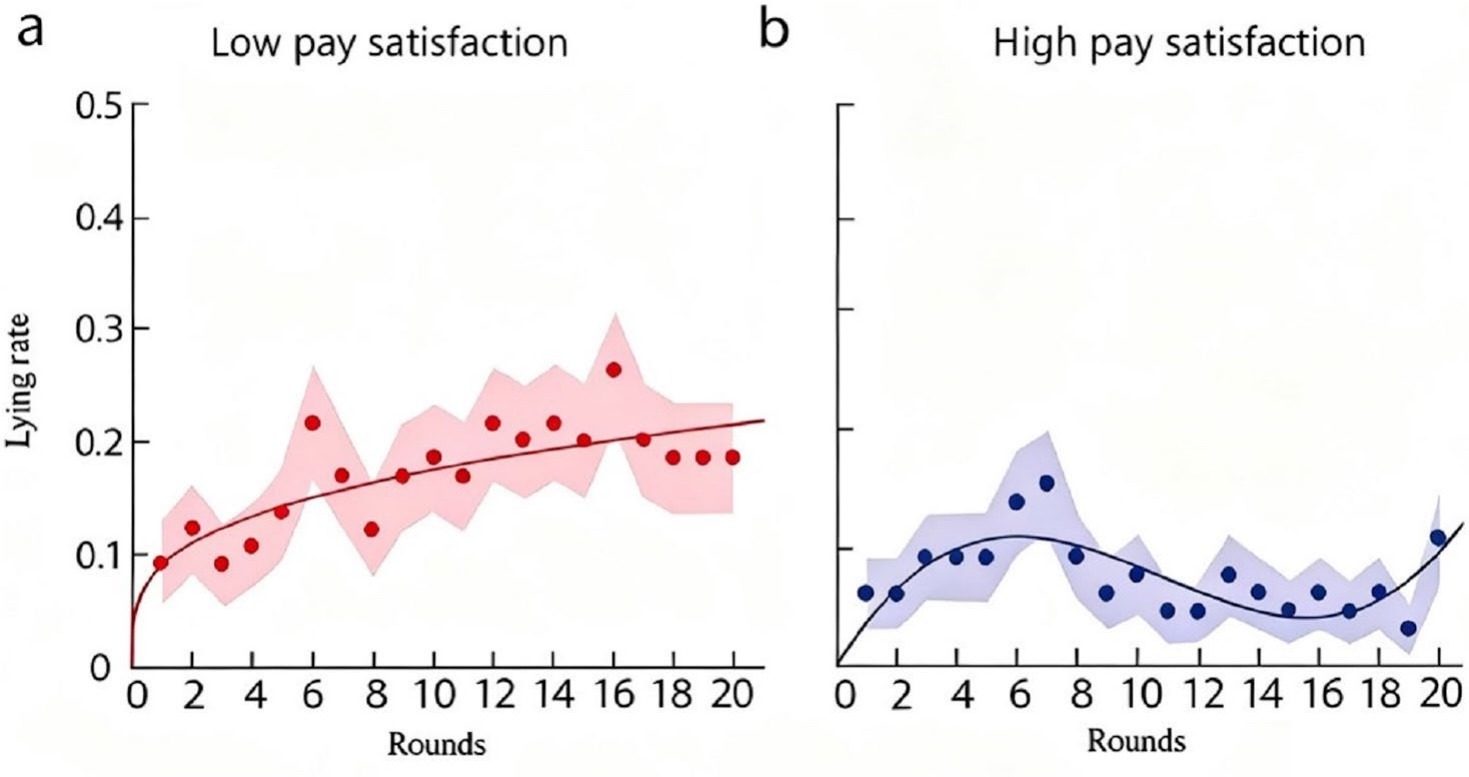
Results of Study 2. Solid dots represent the lying rate across rounds. The shaded area represents the standard deviation of the lying rate after 1000 random samplings. Panel (a) depicts results for the low pay satisfaction group, where the lying rate increases with increasing rounds (*R* ^2^ = 0.59, *χ* ^2^_(18)_ = 0.56, *p* = 1.000). Panel (b) depicts results for the high pay satisfaction group, where the lying rate first increases and then decreases with increasing rounds (with a sharp spike in the last round, Round 20) (*R* ^2^ = 0.49, *χ* ^2^_(18)_ = 0.43, *p* = 1.000).

Curve‐fitting analyses revealed that for the low and high pay satisfaction groups, the power curve and cubic model were the best‐fitting curves for the “lying rate‐rounds” condition (Supporting Information S2). The distributed calculation results are as follows. Figure 4 illustrates how the lying rate changed across rounds. As shown in Figure 4a, for the low pay satisfaction group, the lying rate increased as rounds progressed and followed a power function distribution (*R* ^2^ = 0.59, *χ* ^2^_(18)_ = 0.56, *p* = 1.00), confirming the presence of the moral slippery slope effect. As shown in Figure 4b, for the high pay satisfaction group, the lying rate first increased and then decreased as rounds progressed, with a sudden increase in the final round (round 20). This pattern followed a cubic function distribution (*R* ^2^ = 0.49, *χ* ^2^_(18)_ = 0.43, *p* = 1.00), indicating that the moral slippery slope effect disappeared in the high pay satisfaction group, though it was accompanied by a final‐round lying effect.

#### 3.2.4 GEE Analyses

We used a GEE to examine how the number of lies varied across participants in different pay satisfaction groups under the accumulative pay condition. Results showed a significant main effect of group (Wald *χ* ^2^_(1)_ = 12.80, *p* < 0.001), no significant main effect of rounds (Wald *χ* ^2^_(19)_ = 23.81, *p* = 0.204), and no significant interaction between pay satisfaction group and round (Wald *χ* ^2^_(19)_ = 18.55, *p* = 0.486).

Furthermore, we conducted an additional GEE analysis that included self‐reported pay satisfaction scores as covariates, focusing on changes in the number of lies between high and low pay satisfaction groups at rounds 0, 5, 10, 15, and 20. Results revealed a significant main effect of rounds (Wald *χ* ^2^_(4)_ = 77.52, *p* < 0.001), a significant main effect of group (Wald *χ* ^2^_(1)_ = 3.76, *p* < 0.001), and no significant effect of self‐reported pay satisfaction (Wald *χ* ^2^_(1)_ = 1.13, *p* < 0.001). Importantly, the interaction between rounds and group was significant (Wald *χ* ^2^_(4)_ = 16.80, *p* < 0.01). The results of a separate effect analysis indicated that, compared to the high pay satisfaction group, the low pay satisfaction group showed a larger discrepancy in measured lying frequency from the first round as the experiment progressed (Supporting Information S3).

#### 3.2.5 Moderating Effect of Pay Satisfaction

Results of the grouping regression analyses are presented in Table 2. The non‐standardized *Z*‐test results showed *Z* = −4.38 (*Z* < −1.96), indicating that pay satisfaction significantly moderated the relationship between rounds and lying rate (see Table 2).

**TABLE 2:** Study 2: Grouping regression test of pay satisfaction.

Group		Non‐standardized		Standardized
		*B*	SE	
Low‐group	(Constant)	10.182	1.666	0.709
	Round	0.592	0.139	
High‐group	(Constant)	9.648	1.393	−0.377
	Round	−0.201	0.116	

### 3.3 Discussion

Study 2 results demonstrate that under the 20‐round accumulative pay condition, pay satisfaction significantly moderates the moral slippery slope effect. Specifically, individuals with low pay satisfaction engaged in more unethical behaviors, and the slippery slope effect persisted. In contrast, those with high pay satisfaction exhibited less unethical behavior, and the upward trend of the slippery slope effect showed a phased decline. These findings provide preliminary support for Hypothesis 2.

Notably, although the upward trend of the slippery slope effect among participants in the high pay satisfaction group showed a decline, the lying rate (a key indicator of the effect) increased again in the final rounds. This pattern aligns with the “cheat‐at‐the‐end effect” identified in prior research (Effron et al. 2015). Examples of this effect include consumers increasing spending on the final day of mall discount events and a surge in cheating as exam submission deadlines approach (McKenzie et al. 2016). The presence of this effect in our study suggests that some participants in the high pay satisfaction group might still be unsatisfied with their current pay.

Previous studies have shown that pay satisfaction is shaped by an individual's perception of the gap between ideal and actual pay, as well as personal traits differences (Gneezy 2005; Gneezy et al. 2013). However, the manipulation of pay satisfaction in Study 2 relied solely on setting a fixed basic pay level, which failed to reflect the relationship between ideal pay and actual pay. This relationship is a key driver of pay satisfaction. Another limitation is the imbalanced gender distribution: male participants accounted for a much smaller proportion than female participants. To address these gaps, Study 3 aimed to balance the gender distribution and incorporated participants' ideal pay into the pay satisfaction manipulation, further exploring how pay satisfaction moderates the disappearance of the moral slippery slope effect.

## 4 Study 3

Study 3 was designed to address the limitations of Study 2. During recruitment, Study 3 first collected data on each participant's ideal pay. Both groups in this study were provided with a basic pay tailored to their ideal pay: specifically, the basic pay for the low pay satisfaction group was set at 10% of their ideal pay, while that for the high pay satisfaction group was set at 60% of their ideal pay.

### 4.1 Method

#### 4.1.1 Participants

Study 3 recruited 128 college students, primarily motivated by monetary pay. The sample included 63 females and 65 males, with a mean age of 21.20 years (SD = 1.99). Prior to the formal dice‐rolling task, all participants reported their ideal pay (range = 8–13 Yuan) via the E‐Prime interface. They were explicitly informed that pay for diligent task completion included two components: basic pay and additional task pay. The basic pay was set at 10%–60% of their reported ideal pay, while the additional task pay consisted of total earnings from the 20‐round dice‐rolling task. Inclusion criteria required participants to have normal or corrected‐to‐normal vision, be right‐handed, be free from fatigue, and be naive to similar studies. Written informed consent was obtained from all participants before the study commenced.

#### 4.1.2 Study Design

A 2 (pay satisfaction: high vs. low) × 20 rounds mixed‐factorial design was used. Pay satisfaction served as the between‐subjects variable, with 64 participants assigned to each condition. Rounds served as the within‐subjects variable (20 total rounds). The dependent variable, unethical behavior, was operationalized as whether participants lied in each round of the dice‐rolling task.

#### 4.1.3 Paradigm

The paradigm used in Study 3 was identical to that in Study 1.

#### 4.1.4 Procedure

Participants in both the high and low pay satisfaction conditions received basic pay, with the specific amount determined by their previously reported ideal pay.

The study procedure was as follows. Before the task began, participants received condition‐specific instructions. Low pay satisfaction condition: “This task uses an accumulative pay structure. Please monitor your accumulated pay throughout the task. Your basic pay for this task is 10% of your reported ideal pay; you can also earn additional rewards in the upcoming 20‐round dice‐rolling task, which will be included in your final pay.” High pay satisfaction condition: “This task uses an accumulative pay structure. Please monitor your accumulated pay throughout the task. Your basic pay for this task is 60% of your reported ideal pay; you can also earn additional rewards in the upcoming 20‐round dice‐rolling task, which will be included in your final pay.” Participants then completed the 20‐round dice‐rolling task, with lying behavior recorded for each round (consistent with Study 1 and 2).

#### 4.1.5 Data Analysis

Sample size determination: The sample size was determined based on the effect size observed in Study 2. An a priori power analysis (conducted using G*Power; Faul et al. 2007) indicated that a sample of 128 participants would suffice to detect a target effect size of *d* = 0.50 under standard criteria (*α* = 0.05, 1 − *β* = 0.80, two‐tailed).

Statistical analyses for the manipulation check of self‐reported pay satisfaction, the correlation between self‐reported pay satisfaction and number of lies, curve fitting, and GEE followed the same procedures as in Study 2.

The distributed calculation using MATLAB 13.5 was as follows: power function fitting (Equation 1) was applied to model the change in lying rate across rounds for the low pay satisfaction condition. Quadratic function fitting (Equation 2) was applied to model the change in lying rate across rounds for the high pay satisfaction condition.

The moderating role of pay satisfaction in the relationship between rounds and lying rate was analyzed using the same approach as in Study 2.

### 4.2 Results

#### 4.2.1 Self‐Reported Pay Satisfaction Manipulation Check

Nonparametric Mann–Whitney *U* tests revealed significant differences in self‐reported pay satisfaction between the two groups across all rounds (*Z*s < −3.67, *p*s < 0.001, *d*s > 1.033). Self‐reported pay satisfaction of the low pay satisfaction group (0 rounds: *M ±* SD = 2.27 ± 1.37, 5 rounds: *M ±* SD = 2.78 ± 1.41, 10 rounds: *M ±* SD = 3.09 ± 1.49, 15 rounds: *M* ± SD = 3.47 ± 1.25, 20 rounds: *M ±* SD = 4.11 ± 1.50) was consistently lower than that of the high pay satisfaction group (0 rounds: *M ±* SD = 5.03 ± 1.27, 5 rounds: *M ±* SD = 4.52 ± 1.30, 10 rounds: *M ±* SD = 4.83 ± 1.27, 15 rounds: *M ±* SD = 5.23 ± 1.05, 20 rounds: *M ±* SD = 5.95 ± 1.03). These results confirm the effectiveness of the pay satisfaction manipulation in this study.

We further analyzed correlations between each group's self‐reported pay satisfaction at rounds 0, 5, 10, 15, and 20 and their lying behaviors at the corresponding stages. Results showed that low pay satisfaction was significantly correlated with lying behaviors (*r* = 0.13, *p* = 0.024), whereas high pay satisfaction was not significantly correlated with lying behaviors (*r* = 0.07, *p* = 0.271).

#### 4.2.2 Lying Frequency and Normality Tests Across Groups

Kolmogorov–Smirnov tests for normality revealed the following results: the low pay satisfaction group failed to reject the null hypothesis of normality (*p* = 0.200), indicating its lying frequency distribution conformed to a normal distribution; the high pay satisfaction group rejected the null hypothesis (*p* = 0.028, *p* < 0.05), indicating its lying frequency distribution deviated from a normal distribution. Furthermore, a Mann–Whitney *U* test showed a significant difference in total lying frequency across the 20 rounds between the two groups (*U* = 2.000, *p* < 0.001). Specifically, the low pay satisfaction group had a significantly higher total lying frequency (*M* = 3.86, SD = 4.07) than the high pay satisfaction group (*M* = 1.06, SD = 1.49).

Lying frequencies of the two groups across the 20 rounds are presented in Table 3. Consistent with the total frequency results, the low pay satisfaction group had a higher overall lying frequency than the high pay satisfaction group. At the single‐round level, the low pay satisfaction group had a maximum total lying frequency of 21 times, whereas the high pay satisfaction group had a maximum of 7 times.

**TABLE 3:** Study 3: The lying frequency of the two groups.

Low pay satisfaction group			High pay satisfaction group		
Number of lies	Frequency	Effective percentage	Number of lies	Frequency	Effective percentage
6	1	5.00	1	1	5.00
8	1	5.00	2	7	35.00
9	1	5.00	3	4	20.00
10	3	15.00	4	2	10.00
11	1	5.00	5	3	15.00
12	2	10.00	6	2	10.00
13	3	15.00	7	1	5.00
14	2	10.00	—	—	—
15	2	10.00	—	—	—
16	1	5.00	—		—
17	1	5.00	—	—	—
18	1	5.00	—	—	—
21	1	5.00	—	—	—

#### 4.2.3 Model Analysis

**FIGURE 5:**
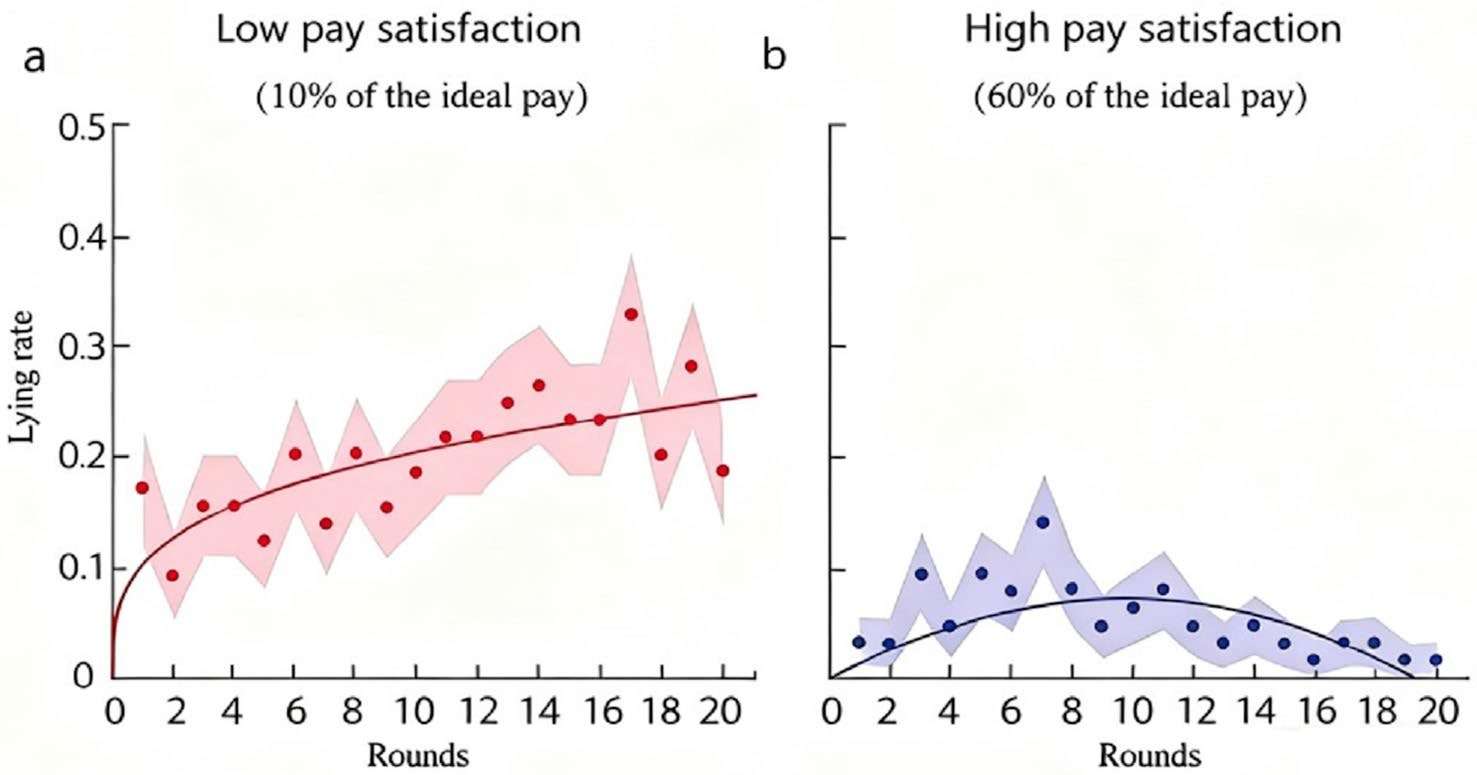
Results of Study 3. Panel (a) depicts results for the low pay satisfaction group (10% of ideal pay), where the lying rate increases with increasing rounds (*R* ^2^ = 0.48, *χ* ^2^_(18)_ = 0.77, *p* = 1.000). Panel (b) depicts results for the high pay satisfaction group (60% of ideal pay), where the lying rate first increases and then decreases with increasing rounds (*R* ^2^ = 0.63, *χ* ^2^_(18)_ = 0.55, *p* = 1.000).

Curve‐fitting analyses revealed that for the low and high pay satisfaction groups, the power curve and quadratic model were the best‐fitting curves for the “lying rate–rounds” relationship condition (Supporting Information S3). The distributed calculation results are as follows. Figure 5 illustrates changes in the lying rate across rounds in Study 3. As shown in Figure 5a, for the low pay satisfaction group, the lying rate increased as rounds progressed and followed a power function distribution (*R* ^2^ = 0.48, *χ* ^2^_(18)_ = 0.77, *p* = 1.00), indicating the presence of the moral slippery slope effect. As shown in Figure 5b, for the high pay satisfaction group, the lying rate first increased and then decreased with increasing rounds. Unlike Study 2, there was no sudden increase in the lying rate in the final round (Round 20). This pattern followed a quadratic function distribution (*R* ^2^ = 0.63, *χ* ^2^_(18)_ = 0.55, *p* = 1.00), indicating that the moral slippery slope effect disappeared in the high pay satisfaction group, and the final‐round surge in unethical behavior observed in Study 2 was absent.

#### 4.2.4 GEE Analyses

We used a GEE to examine variations in the number of lies among participants in different pay satisfaction groups under the accumulative pay condition. Results showed a significant main effect of group (Wald *χ* ^2^_(1)_ = 30.46, *p* < 0.001), and a significant interaction between group and rounds (Wald *χ* ^2^_(19)_ = 32.61, *p* < 0.05).

Furthermore, we conducted an additional GEE analysis that included self‐reported pay satisfaction scores as covariates, focusing on changes in the number of lies between high and low pay satisfaction groups at rounds 0, 5, 10, 15, and 20. Results revealed a significant main effect of rounds (Wald *χ* ^2^_(4)_ = 80.404, *p* < 0.001), a significant main effect of group (Wald *χ* ^2^_(1)_ = 11.009, *p* < 0.001), a significant effect of self‐reported pay satisfaction (Wald *χ* ^2^_(1)_ = 4.00, *p* < 0.05), and a significant interaction between rounds and group (Wald *χ* ^2^_(4)_ = 37.44, *p* < 0.001). The results of a separate effect analysis indicate that compared to the high pay satisfaction group, the low pay satisfaction group showed a larger discrepancy in the measured number of lies from the first round as the experiment progressed (Supporting Information S3).

#### 4.2.5 Moderating Effect of Pay Satisfaction

Results of the grouping regression analyses are presented in Table 4. Non‐standardized *Z*‐test results showed *Z* = −4.82 (*Z* < 1.96), indicating that pay satisfaction significantly moderated the relationship between rounds and lying rate.

**TABLE 4:** Study 3: Grouping regression test of pay satisfaction.

Group		Non‐standardized		Standardized
		*B*	SE	
Low‐group	(Constant)	12.462	2.24	0.689
	Round	0.755	0.187	
High‐group	(Constant)	8.284	1.32	−0.527
	Round	−0.290	0.11	

### 4.3 Discussion

Study 3 results demonstrate that under the 20‐round accumulative pay condition, pay satisfaction significantly moderates the moral slippery slope effect. Specifically, individuals with lower pay satisfaction engaged in more unethical behaviors, and the moral slippery slope effect persisted. In contrast, those with higher pay satisfaction exhibited fewer unethical behaviors, and the moral slippery slope effect disappeared.

Notably, compared to Study 2, Study 3 refined the pay satisfaction manipulation by tailoring basic pay to each participant's self‐reported ideal pay. With this more precise manipulation, the high pay satisfaction group no longer exhibited a resurgence of unethical behavior in the final rounds. This finding indicates that when accumulative pay aligns with participants' ideal pay, the pay level adequately meets their expectations, eliminating the motivation to engage in increased unethical behavior toward the task's end.

## 5 General Discussion

To resolve the conflicting findings in prior research on the moral slippery slope effect, Study 1 was designed to examine whether pay type (accumulative vs. non‐accumulative) directly shapes the trajectory of unethical behavior. Results established this relationship: under non‐accumulative pay, the moral slippery slope effect persisted, with lying rates increasing steadily across rounds. In contrast, under accumulative pay, the effect diminished over time. Accumulative pay not only represents accumulated economic gains but also shapes individuals' psychological perceptions and decision‐making choices (Peter 2023; Kanu et al. 2023; Olafsen et al. 2024). By ensuring predictable and accumulable earnings, accumulative pay enhances “earnings satisfaction,” enabling participants to evaluate whether their gains align with expectations (Effron et al. 2015).

Study 1 verified that accumulative pay weakens the moral slippery slope effect. Building on Simon's (1978) Principle of Satisfaction and empirical evidence from Tang and Chiu (2003), we further hypothesized that “pay satisfaction” may function as the core mechanism. Extending Study 1, Study 2 manipulated pay satisfaction via fixed basic pay levels. Results revealed that low pay satisfaction sustained the moral slippery slope effect, with unethical behavior escalating across rounds, consistent with prior research linking pay dissatisfaction to reduced employee engagement, higher turnover, and increased fraudulent behavior (Greenberg 1993; John et al. 2014). Under high pay satisfaction, the effect was largely mitigated. However, a residual “cheat‐at‐the‐end effect” emerged, marked by a sudden surge in lying during the final round. Simon's (1978) Principle of Satisfaction posits that decision‐makers cease pursuing gains once subjective expectations are met, rather than striving for optimal outcomes. This sudden surge in lying thus partly indicates that the assigned pay failed to satisfy participants. As noted by researchers, pay satisfaction often stems not from absolute levels but from the perceived gap between actual and ideal outcomes (John et al. 2014). Based on this, we speculate that the “cheat‐at‐the‐end effect” in Study 2 arose because the fixed high basic pay did not fully align with all participants' subjective ideal pay, leading to a surge in unethical behavior in the final round as a means of compensating for unmet expectations.

Study 3 manipulated pay satisfaction by setting basic pay based on the proportion of participants' ideal pay. Results confirmed that under high pay satisfaction, unethical behavior followed a “rise‐then‐decline” pattern: the moral slippery slope effect was fully eliminated, with no end‐of‐task surge in unethical acts. Empirically, higher pay satisfaction correlates with fewer unethical behaviors (Hofmann et al. 2009; Chen et al. 2014; Reichheld 1993), and Veldhuizen (2013) demonstrated that pay satisfaction reduces unethical acts such as accepting bribes. When accumulative pay matches ideal expectations, the absence of a gap between actual and desired outcomes resolves the psychological conflict between self‐interest and honesty, a conflict identified as the driver of the moral slippery slope effect (Banaji et al. 2003; Gino and Bazerman 2009; Mazar et al. 2008; Moore et al. 2012; Kouchaki et al. 2018; Yang et al. 2015).

The moral cleansing effect posits that early minor transgressions heighten sensitivity to one's moral self‐concept, prompting subsequent restraint. This is particularly evident among participants satisfied with their pay, who become more attuned to the costs of lying for preserving their moral image (Zhong and Liljenquist 2006; Yoel et al. 2013; Fan, Sun, et al. 2025; Fan, Zhou, et al. 2025). For high‐satisfaction participants, the perceived moral risks and consequences of lying (e.g., damaging one's moral image) grew increasingly salient over time, enhancing their emotional processing and cognitive control (Dupont et al. 2023; Sun et al. 2024). Their reduced unethical behavior thus likely stemmed from a desire to mitigate moral risks and meet social expectations, rather than mere pursuit of personal gain.

### 5.1 Contributions and Implications

#### 5.1.1 Contributions

First, our study clarifies the boundary conditions of the moral slippery slope effect and resolves inconsistencies in prior research. While previous studies have consistently confirmed the existence of the moral slippery slope effect (Moore et al. 2012; Tenbrunsel and Messick 2004; Adelman et al. 2021; Anderson et al. 2024), they have not systematically explored the contextual factors that constrain or activate the effect. Our research demonstrates that the persistence of the effect hinges critically on pay type: it endures under non‐accumulative pay but diminishes under accumulative pay. This finding provides more targeted insights into understanding when unethical behavior in organizations subsides and when it escalates.

Second, our study identifies pay satisfaction as the core mechanism that weakens the moral slippery slope effect under accumulative pay, addressing unresolved issues in existing research. It clarifies that it is not accumulative pay itself, but pay satisfaction, that moderates the moral slippery slope effect. This conclusion bridges the gap between compensation structures and the psychological processes shaping moral behavior. By focusing on key psychological variables, it advances understanding of how compensation systems influence ethical decision‐making. Specifically, low pay satisfaction increases individuals' susceptibility to external temptations, thereby sustaining the moral slippery slope effect (John et al. 2014; Barnes et al. 2015; Kouchaki et al. 2018). Conversely, high pay satisfaction enhances individuals' self‐regulation, self‐control, and moral responsibility, thereby reducing or eliminating the effect (Hofmann et al. 2018; Heneman and Schwab 1972; Shalvi et al. 2011).

Finally, our study enriches the theoretical content through which pay satisfaction moderates the moral slippery slope effect and deepens understanding of the link between subjective evaluation of pay and unethical behavior. Integrating Nisan's Moral Balance Theory (Nisan and Horenczyk 1990; Nisan 1991) and the moral cleansing effect (Zhong and Liljenquist 2006; Zhang and Du 2023), it reveals that high pay satisfaction resolves the psychological conflict between self‐interest and honesty, thereby inhibiting the escalation of unethical behavior. This pathway connects macro‐level pay structures with micro‐level psychological mechanisms, providing a more comprehensive theoretical framework for understanding unethical behavior in long‐term contexts.

#### 5.1.2 Implications

The findings of this study provide specific guidance for organizational practice and policy‐making, based on the link between pay and ethical behavior.

In organizational practice, unethical behavior can be mitigated through two approaches: first, for roles with high ethical risks (e.g., finance, auditing), prioritize accumulative pay structures such as tiered performance incentives to create conditions for pay satisfaction to take effect; second, regularly survey employees' ideal pay expectations, enhance satisfaction by narrowing the gap between actual and expected earnings, and reduce unethical behavior stemming from unmet expectations.

For policy‐making, enterprises can be guided to incorporate pay satisfaction into internal ethical risk assessments, especially in high‐risk industries such as finance and auditing. This would urge enterprises to prioritize pay equity and employee demands, reducing the risk of moral slippage at its source.

### 5.2 Limitations and Future Directions

This study has several limitations that warrant attention in future research.

Firstly, we mainly focused on interest‐driven motives when selecting participants, as unethical behaviors are often economically incentivized. We employed the spontaneous deception paradigm, a classic approach in moral decline research, and specifically observed lying behavior in a dice‐rolling task with monetary rewards. Future research could explore whether similar effects emerge among individuals motivated by nonfinancial factors, such as intrinsic rewards or nonmonetary incentives.

Second, our research relied on a standardized dice‐rolling task to examine the moral slippery slope effect at the group level, focusing on round‐by‐round trends in lying rates and differences between conditions. However, it lacks in‐depth individual‐level analysis. Such analysis, which includes tracking changes in individual participants' lying behavior or linking individual traits like moral sensitivity to unethical acts, would better capture individual differences in the quantity and severity of unethical behavior. Future studies could employ individual‐level tasks, such as the send–receive task or the matrix task (Gerlach et al. 2019), to explore whether individual pay satisfaction moderates the frequency and severity of such unethical behavior.

Third, unethical behavior was measured using binary coding (0 = no lie, 1 = lie). While this effectively captures the incidence of lying and supports analyses of round‐by‐round rates, which is consistent with our core goal of testing how pay type and pay satisfaction influence lying, it fails to capture the “degree of dishonesty,” such as the magnitude of exaggerated dice rolls. This limits our ability to reflect the “gradual escalation in unethical severity,” a nuanced feature of the moral slippery slope effect. Future research could incorporate continuous measures of unethical severity, such as calculating discrepancies between reported and actual values, to address this gap.

Fourth, inconsistencies emerged between Study 1 and Study 2 (low pay satisfaction condition), both of which investigated the moral slippery slope effect under accumulative pay without basic pay. In Study 2, participants with low pay satisfaction recorded their gains and satisfaction every five rounds. This frequent tracking may have induced more unethical behavior and strengthened the moral slippery slope effect. In contrast, Study 1 showed a weaker effect, likely because accumulative pay was less salient in its task design. Future research should refine these manipulations and account for task design factors to clarify the effect more precisely.

Additionally, while our experimental design aimed to identify how pay satisfaction affects unethical behavior, future studies could explore the reciprocal relationship between the two. Using longitudinal designs or developing metrics to track how unethical behavior influences pay and satisfaction over time would enable a more dynamic framework. This framework, incorporating bidirectional causality, may illuminate their ongoing interaction over time.

Moreover, although we found that high pay satisfaction diminishes the moral slippery slope effect, the underlying mechanisms driving this attenuation remain unexplored. Future research should investigate how high pay satisfaction mitigates the ethical tension between honesty and profit‐seeking.

Finally, our study was conducted in a controlled laboratory setting, which may not fully reflect the complexity of real‐world organizational environments. Deceptive behavior can elicit psychological conflicts and moral self‐awareness, accompanied by physiological changes (Eisenberg 2000; Qu et al. 2020). Future research could use tools such as polygraphs or video recordings to capture physiological and behavioral responses. It could further examine how employees' pay satisfaction impacts ethical behavior in real‐world workplaces, developing a more comprehensive understanding of moral decline in actual organizations.

## 6 Conclusion

In summary, this study's three experiments yield two core findings that delineate the factors shaping the moral slippery slope effect. First, accumulative pay serves as a key contextual prerequisite for the attenuation of the moral slippery slope effect: under non‐accumulative pay, the effect persisted consistently, with lying rates rising steadily as rounds increased; under accumulative pay, however, the effect did not persist, and instead lying rates first increased and then decreased, with the moral slippery slope effect subsiding in the later rounds. Second, building on the above finding about accumulative pay, pay satisfaction acts as a significant moderator of the moral slippery slope effect under this pay structure: specifically, participants with high pay satisfaction demonstrated lower lying rates overall, the moral slippery slope effect disappeared, with no upward trend in unethical behavior observed in subsequent rounds; conversely, those with low pay satisfaction exhibited higher lying rates, and the moral slippery slope effect remained persistent. Practically, in pay design contexts, enhancing pay satisfaction serves as a strategic means to curb the moral slippery slope effect. By integrating considerations of the nature of pay accumulation and individuals' satisfaction needs into pay system design, organizations can aid in mitigating the risks of unethical escalation, fostering a more ethically compliant organizational environment.

## Funding

This work was supported by Zhejiang Provincial Philosophy and Social Sciences Planning Project (grant number 25NDJC001ZD).

## Ethics Statement

This research was conducted in accordance with the Declaration of Helsinki. The Ethics Committee of the School of Psychology, Zhejiang Normal University, approved the study. The participants interested in participating read the informed consent, which contained the objectives, benefits, and risks of participation; those who agreed to participate in the study gave their informed consent, accepting to continue with the survey. The participants were informed that their responses to the task would be anonymous and confidential, and the data collected would be used for academic research only.

## Conflicts of Interest

The authors declare no conflicts of interest.

## Supporting information


**Data S1:** Supporting Information.
