# Supplementary material for: How Does Less Unethical Behavior Happen? The Moderating Role of Pay Satisfaction on the Disappearance of the Moral Slippery Slope Effect
Source: Psych J. 2025 Dec 17;15(1):e70071. doi: 10.1002/pchj.70071 (PMC12856240; doi:10.1002/pchj.70071)
Supplement: Supplementary file 1 — Data S1: Supporting Information. [file PCHJ-15-e70071-s001.docx]

**S1 Study 1**

**S1.1 Participants**


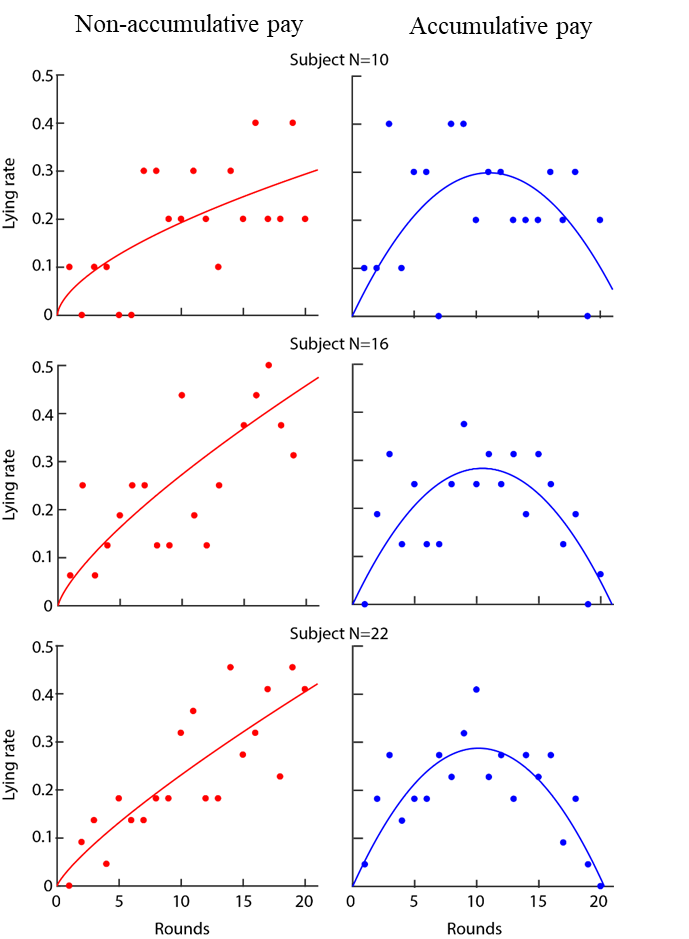


Figure S1 Lying rate against rounds as the subject number is 10, 16, and 22, showing that the results of Study 1 are robust.

**S1.2** **Curve Estimation**

Curve fitting results indicated that under the non-accumulative pay condition, the adjusted R^2^ for the power curve was 0.84, demonstrating that the power curve had the best fit with the scatter plot under this condition. Thus, the power curve was selected as the curve model for the “lying rate–rounds” relationship in the non-accumulative pay condition (Table S1). Under the accumulative pay condition, only the cubic and quadratic models were statistically significant (*p* < 0.001). Further analysis revealed that the regression coefficients in the cubic model were non-significant and exceeded the acceptable range in terms of magnitude. Therefore, the quadratic model was determined to be the best-fitting curve for the “lying rate–rounds” relationship under the accumulative pay condition (Table S2).

Table S1 Main Statistics for curve fitting under non-accumulative pay condition

| Model | Model statistics | | | |  |
| --- | --- | --- | --- | --- | --- |
|  | adj *R^2^* | *SE* | *F* | *p* | |
| Linear | 0.78 | 4.95 | 66.48 | 0.001 | |
| Logarithmic | 0.68 | 5.88 | 42.00 | 0.001 | |
| Quadratic | 0.78 | 5.09 | 31.57 | 0.001 | |
| Cubic | 0.75 | 5.24 | 19.83 | 0.001 | |
| Compound | 0.79 | 0.26 | 70.23 | 0.001 | |
| Power | 0.84 | 0.29 | 98.09 | 0.001 | |
| Growth | 0.79 | 0.26 | 70.23 | 0.001 | |
| Exponential | 0.79 | 0.26 | 70.23 | 0.001 | |

Table S2 Main Statistics for curve fitting under accumulative pay condition

| Model | Main statistics | | | |  |
| --- | --- | --- | --- | --- | --- |
|  | adj *R^2^* | *SE* | *F* | *p* | |
| Quadratic | 0.67 | 2.06 | 20.60 | 0.001 | |
| Cubic | 0.66 | 2.13 | 13.05 | 0.001 | |

**S1.3 Generalized Estimation Equation**

For Model 1, a Generalized Estimation Equation (GEE) was constructed, with fixed effects including round (20 rounds total) and pay type (accumulative vs. non-accumulative conditions), and a random effect of participant ID; the dependent variable was whether each participant lied in each round (see Table S3).

Table S3 Generalized estimation equation

| Variable |  | Model1 | | | | |
| --- | --- | --- | --- | --- | --- | --- |
|  |  | *B(95%CI)* | | Wald *X^2^* | *P* | |
| accumulative pay |  | |  | | |  |
| 20 | 0.03 (-0.06, 0.12) | | 0.34 | | | 0.562 |
| 19 | 0 (-0.07, 0.07) | | 0.01 | | | 1.000 |
| 18 | 0.16 (0.02, 0.30) | | 5.10 | | | 0.024 |
| 17 | 0.08(-0.03, 0.19) | | 1.89 | | | 0.169 |
| 16 | 0.21(0.08, 0.34) | | 10.13 | | | 0.001 |
| 15 | 0.26(0.11, 0.42) | | 10.67 | | | 0.001 |
| 14 | 0.18(0.06, 0.31) | | 8.58 | | | 0.003 |
| 13 | 0.26(0.11, 0.42) | | 10.67 | | | 0.001 |
| 12 | 0.24(0.08, 0.39) | | 9.13 | | | 0.003 |
| 11 | 0.24(0.08, 0.39) | | 9.13 | | | 0.003 |
| 10 | 0.37(0.20, 0.54) | | 18.08 | | | 0.001 |
| 9 | 0.24(0.08, 0.39) | | 9.13 | | | 0.003 |
| 8 | 0.24(0.08, 0.39) | | 9.13 | | | 0.003 |
| 7 | 0.18(0.04, 0.33) | | 6.36 | | | 0.012 |
| 6 | 0.21(0.08, 0.34) | | 10.13 | | | 0.001 |
| 5 | 0.21(0.08, 0.34) | | 10.13 | | | 0.001 |
| 4 | 0.11(0.01, 0.20) | | 4.47 | | | 0.034 |
| 3 | 0.21(0.08, 0.34) | | 10.13 | | | 0.001 |
| 2 | 0.16(0.02, 0.30) | | 5.10 | | | 0.024 |
| 1 | 0 | | - | | | - |
| non-accumulative pay |  | |  | | |  |
| 20 | 0.34(0.19, 0.49) | | 19.76 | | | 0.001 |
| 19 | 0.32(0.17, 0.46) | | 17.54 | | | 0.001 |
| 18 | 0.18(0.06, 0.31) | | 8.58 | | | 0.003 |
| 17 | 0.32(0.15, 0.48) | | 14.10 | | | 0.001 |
| 16 | 0.26(0.12, 0.40) | | 13.57 | | | 0.001 |
| 15 | 0.21(0.06, 0.36) | | 7.70 | | | 0.006 |
| 14 | 0.34(0.19, 0.49) | | 19.76 | | | 0.001 |
| 13 | 0.16(0.04, 0.27) | | 7.13 | | | 0.008 |
| 12 | 0.13(-0.02, 0.28) | | 3.00 | | | 0.083 |
| 11 | 0.18(0.04, 0.33) | | 6.36 | | | 0.012 |
| 10 | 0.24(0.08, 0.39) | | 9.13 | | | 0.003 |
| 9 | 0.11(0.01, 0.20) | | 4.47 | | | 0.034 |
| 8 | 0.13(0.02, 0.24) | | 5.76 | | | 0.016 |
| 7 | 0.16(0.04, 0.27) | | 7.13 | | | 0.008 |
| 6 | 0.08(-0.03, 0.19) | | 1.89 | | | 0.169 |
| 5 | 0.08(-0.03, 0.19) | | 1.89 | | | 0.169 |
| 4 | 0.03(-0.03, 0.08) | | 1.03 | | | 0.311 |
| 3 | 0.05(-0.05, 0.15) | | 1.03 | | | 0.311 |
| 2 | 0.05(-0.05, 0.15) | | 1.03 | | | 0.311 |
| 1 | 0^a^ | | - | | | - |

**S2 Study 2**

**S2.1 Curve Estimation**

Curve fitting results indicated that in the high pay satisfaction group, the adjusted R² of the power curve was 0.45, yielding the best fit for this group. In the low pay satisfaction group, the adjusted R² of the cubic model was 0.49, which provided the best fit for this group (see Table S4).

Table S4 Main Statistics for curve fitting about two groups

|  | curve fitting | Main statistics | | | |  |
| --- | --- | --- | --- | --- | --- | --- |
|  |  | adj *R^2^* | *SE* | *F* | *p* | |
| low-group | Pow | 0.45 | 2.33 | 6.23 | 0.005 | |
| high-group | Cub | 0.49 | 3.53 | 10.13 | 0.001 | |

**S2.2 Generalized Estimation Equation**

For Model 2, a Generalized Estimation Equation (GEE) was constructed via fixed effects including round (20 rounds total) and pay satisfaction group (high vs. low), plus a random effect of participant ID; the dependent variable was whether each participant lied in each round (see Table S5).

Table S5 Generalized estimation equation in 20 rounds

| Variable | Model2 | | |
| --- | --- | --- | --- |
|  | *B(95%CI)* | *Wald χ ^2^* | *P* |
| low pay satisfaction |  |  |  |
| 20 | 0.11(-0.01, 0.22) | 3.44 | 0.064 |
| 19 | 0.11(0.00, 0.21) | 4.00 | 0.045 |
| 18 | 0.11(0.00, 0.21) | 4.00 | 0.045 |
| 17 | 0.11(0.00, 0.21) | 4.00 | 0.045 |
| 16 | 0.17(0.05, 0.29) | 7.99 | 0.005 |
| 15 | 0.11(-0.01, 0.22) | 3.44 | 0.064 |
| 14 | 0.12(0.01, 0.24) | 4.26 | 0.039 |
| 13 | 0.11(0.01, 0.20) | 4.78 | 0.029 |
| 12 | 0.12(0.02, 0.22) | 5.81 | 0.016 |
| 11 | 0.08(-0.01, 0.17) | 2.90 | 0.088 |
| 10 | 0.09(-0.03, 0.21) | 2.33 | 0.127 |
| 9 | 0.08(-0.03, 0.18) | 1.98 | 0.159 |
| 8 | 0.03(-0.05, 0.12) | 0.50 | 0.478 |
| 7 | 0.08(-0.00, 0.15) | 3.78 | 0.052 |
| 6 | 0.12(0.023, 0.22) | 5.81 | 0.016 |
| 5 | 0.05(-0.05, 0.15) | 0.83 | 0.363 |
| 4 | 0.02(-0.08, 0.11) | 0.11 | 0.739 |
| 3 | 0(-0.10, 0.10) | 0.01 | 1.000 |
| 2 | 0.03(-0.07, 0.14) | 0.34 | 0.563 |
| 1 | 0^a^ | - | - |
| high pay satisfaction |  |  |  |
| 20 | 0.05(-0.05, 0.15) | 0.83 | 0.363 |
| 19 | -0.03(-0.10, 0.04) | 0.67 | 0.412 |
| 18 | 0(-0.06, 0.06) | 0.01 | 1.000 |
| 17 | -0.02(-0.10, 0.06) | 0.14 | 0.705 |
| 16 | 0(-0.09, 0.09) | 0.00 | 1.000 |
| 15 | -0.02(-0.010, 0.06) | 0.14 | 0.705 |
| 14 | 0(-0.09, 0.09) | 0.01 | 1.000 |
| 13 | 0.02(-0.06, 0.10) | 0.14 | 0.705 |
| 12 | -0.02(-0.10, 0.06) | 0.14 | 0.705 |
| 11 | -0.02(-0.10, 0.06) | 0.14 | 0.705 |
| 10 | 0.02(-0.08, 0.11) | 0.11 | 0.739 |
| 9 | 0(-0.07, 0.07) | 0.01 | 1.000 |
| 8 | 0.03(-0.05, 0.12) | 0.50 | 0.478 |
| 7 | 0.09(-0.01, 0.19) | 3.15 | 0.076 |
| 6 | 0.08(-0.02, 0.18) | 2.36 | 0.125 |
| 5 | 0.03(-0.05, 0.12) | 0.50 | 0.478 |
| 4 | 0.03(-0.05, 0.12) | 0.50 | 0.478 |
| 3 | 0.03(-0.05, 0.12) | 0.50 | 0.478 |
| 2 | 0(-0.06, 0.06) | 0.01 | 1.000 |
| 1 | 0^a^ | - | - |

For Model 3, a Generalized Estimation Equation (GEE) was constructed via fixed effects including round stage (5 stages total) and pay satisfaction group (high vs. low), plus a random effect of participant ID; with self-reported pay satisfaction as a covariate, the dependent variable was the number of lies per participant at stages 0, 5, 10, 15, and 20 (see Table S6).

Table S6 Generalized estimation equation at five stages

| Variable | Model3 | | | |  |
| --- | --- | --- | --- | --- | --- |
|  | *B(95%CI)* | *Wald χ ^2^* | *P* | | |
| low pay satisfaction |  |  | |  |  |
| 20 | 4.58(3.29, 5.86) | 48.81 | | 0.001 |  |
| 15 | 3.15 (2.26, 4.04) | 47.82 | | 0.001 |  |
| 10 | 1.85 (1.28, 2.41) | 41.26 | | 0.001 |  |
| 5 | 0.86 (0.53, 1.19) | 26.14 | | 0.001 |  |
| 0 | 0 | - | | - |  |
| high pay satisfaction |  |  | |  |  |
| 20 | 1.05(0.66, 1.44) | 28.32 | | 0.001 |  |
| 15 | 0.94(0.60, 1.28) | 29.66 | | 0.001 |  |
| 10 | 0.70(0.43, 0.98) | 25.37 | | 0.001 |  |
| 5 | 0.30(0.13, 0.46) | 12.94 | | 0.001 |  |
| 0 | 0 | . | | - |  |

**S3 Study 3**

**S3.1 Curve Estimation**

Curve fitting results indicated that in the high pay satisfaction group, the adjusted R² of the power curve was 0.401, yielding the best fit for this group. In the low pay satisfaction group, the adjusted R² of the quadratic model was 0.428, which provided the best fit for this group (Table S7).

Table S7 Main statistics for curve fitting about two groups

|  | curve fitting | Main statistics | | | |  |
| --- | --- | --- | --- | --- | --- | --- |
|  |  | adj *R^2^* | *SE* | *F* | *p* | |
| low-group | Pow | 0.40 | 2.52 | 7.36 | .005 | |
| high-group | Qua | 0. 43 | 4.90 | 8.11 | .003 | |

**S3.2 Generalized Estimation Equation**

For Model 4, a Generalized Estimation Equation (GEE) was constructed via fixed effects including round (20 rounds total) and pay satisfaction group (high vs. low), plus a random effect of participant ID; the dependent variable was whether each participant lied in each round (see Table S8).

Table S8 Generalized estimation equation in 20 rounds

| Variable | Model4 | | | | |
| --- | --- | --- | --- | --- | --- |
|  | *B(95%CI)* | *Wald χ ^2^* | *P* | |  |
| low pay satisfaction |  |  | |  | |
| 20 | 0.03(-0.09, 0.15) | 0.25 | | 0.621 | |
| 19 | 0.14(0.01, 0.27) | 4.57 | | 0.034 | |
| 18 | 0.06(-0.06, 0.18) | 1.02 | | 0.312 | |
| 17 | 0.17(0.03, 0.31) | 5.73 | | 0.028 | |
| 16 | 0.09(-0.05, 0.24) | 1.68 | | 0.201 | |
| 15 | 0.09(-0.06, 0.24) | 1.54 | | 0.222 | |
| 14 | 0.11(-0.03, 0.25) | 2.42 | | 0.125 | |
| 13 | 0.09(-0.05, 0.24) | 1.68 | | 0.206 | |
| 12 | 0.06(-0.07, 0.19) | 0.90 | | 0.342 | |
| 11 | 0.08(-0.05, 0.21) | 1.34 | | 0.253 | |
| 10 | 0.03(-0.08, 0.15) | 0.28 | | 0.602 | |
| 9 | 0.02(-0.12, 0.15) | 0.05 | | 0.822 | |
| 8 | 0.05(-0.08, 0.17) | 0.53 | | 0.474 | |
| 7 | -0.03(-0.14, 0.07) | 0.34 | | 0.564 | |
| 6 | 0.06(-0.08, 0.21) | 0.74 | | 0.393 | |
| 5 | -0.02(-0.14, 0.11) | 0.06 | | 0.812 | |
| 4 | 0(-0.11, 0.11) | 0.01 | | 1.000 | |
| 3 | 0(-0.12, 0.12) | 0.01 | | 1.000 | |
| 2 | -0.08(-0.17, 0.01) | 2.90 | | 0.091 | |
| 1 | 0^a^ | - | | - | |
| high pay satisfaction |  |  | |  | |
| 20 | -0.02(-0.05, 0.02) | 1.02 | | 0.311 | |
| 19 | -0.02(-0.07, 0.04) | 0.34 | | 0.562 | |
| 18 | 0(-0.06, 0.06) | 0.01 | | 1.000 | |
| 17 | 0(-0.06, 0.06) | 0.01 | | 1.000 | |
| 16 | -0.02(-0.07, 0.04) | 0.34 | | 0.563 | |
| 15 | 0(-0.06, 0.06) | 0.01 | | 1.000 | |
| 14 | 0.02(-0.07, 0.04) | 0.20 | | 0.654 | |
| 13 | 0(-0.06, 0.06) | 0.01 | | 1.000 | |
| 12 | 0.02(-0.02, 0.05) | 1.02 | | 0.312 | |
| 11 | 0.05(-0.03, 0.13) | 1.31 | | 0.257 | |
| 10 | 0.03(-0.03, 0.09) | 1.02 | | 0.312 | |
| 9 | 0.02(-0.04, 0.07) | 0.34 | | 0.564 | |
| 8 | 0.05(-0.02, 0.11) | 1.85 | | 0.177 | |
| 7 | 0.11(0.01, 0.21) | 4.79 | | 0.030 | |
| 6 | 0.05(-0.02, 0.11) | 1.85 | | 0.173 | |
| 5 | 0.06(-0.02, 0.15) | 2.07 | | 0.153 | |
| 4 | 0.02(-0.05, 0.08) | 0.20 | | 0.652 | |
| 3 | 0.06(-0.02, 0.15) | 2.07 | | 0.153 | |
| 2 | 0(-0.06, 0.06) | 0.01 | | 1.000 | |
| 1 | 0^a^ | - | | - | |

*Note:* The pay satisfaction is the ideal pay of the advance survey.

For Model 5, a Generalized Estimation Equation (GEE) was constructed via fixed effects including round stage (5 stages total) and pay satisfaction group (high vs. low), plus a random effect of participant ID; with self-reported pay satisfaction as a covariate, the dependent variable was the number of lies per participant at stages 0, 5, 10, 15, and 20 (see Table S9).

Table S9 Generalized estimation equation at five stages

| Variable | Model5 | | | | |
| --- | --- | --- | --- | --- | --- |
|  | *B(95%CI)* | *Wald χ ^2^* | *P* | |  |
| low pay satisfaction |  |  | |  | |
| 20 | 4.58(3.29, 5.86) | 48.81 | | 0.001 | |
| 15 | 3.10 (2.26, 4.04) | 47.82 | | 0.001 | |
| 10 | 1.85 (1.28, 2.41) | 41.26 | | 0.001 | |
| 5 | 0.86 (0.53, 1.19) | 26.14 | | 0.001 | |
| 0 | 0 | - | | - | |
| high pay satisfaction |  |  | |  | |
| 20 | 1.05(0.66, 1.44) | 28.32 | | 0.001 | |
| 15 | 0.94(0.60, 1.28) | 29.66 | | 0.001 | |
| 10 | 0.70(0.43, 0.98) | 25.37 | | 0.001 | |
| 5 | 0.30(0.13, 0.46) | 12.94 | | 0.001 | |
| 0 | 0 | . | | - | |

*Note:* The pay satisfaction is the ideal pay of the advance survey.
